# Seq4SNPs: new software for retrieval of multiple, accurately annotated DNA sequences, ready formatted for SNP assay design

**DOI:** 10.1186/1471-2105-10-180

**Published:** 2009-06-12

**Authors:** Helen I Field, Serena A Scollen, Craig Luccarini, Caroline Baynes, Jonathan Morrison, Alison M Dunning, Douglas F Easton, Paul DP Pharoah

**Affiliations:** 1 Departments of Oncology, University of Cambridge, Cambridge, CB1 8RN, UK; 2 Department of Biochemistry, University of Cambridge, Cambridge, CB2 1QW, UK; 3 Public Health and Primary Care, University of Cambridge, Cambridge, CB1 8RN, UK

## Abstract

**Background:**

In moderate-throughput SNP genotyping there was a gap in the workflow, between choosing a set of SNPs and submitting their sequences to proprietary assay design software, which was not met by existing software. Retrieval and formatting of sequences flanking each SNP, prior to assay design, becomes rate-limiting for more than about ten SNPs, especially if annotated for repetitive regions and adjacent variations. We routinely process up to 50 SNPs at once.

**Implementation:**

We created *Seq4SNPs*, a web-based, walk-away software that can process one to several hundred SNPs given rs numbers as input. It outputs a file of fully annotated sequences formatted for one of three proprietary design softwares: TaqMan's Primer-By-Design FileBuilder, Sequenom's iPLEX or SNPstream's *Autoprimer*, as well as unannotated fasta sequences. We found genotyping assays to be inhibited by repetitive sequences or the presence of additional variations flanking the SNP under test, and in multiplexes, repetitive sequence flanking one SNP adversely affects multiple assays. Assay design software programs avoid such regions if the input sequences are appropriately annotated, so we used *Seq4SNPs *to provide suitably annotated input sequences, and improved our genotyping success rate. Adjacent SNPs can also be avoided, by annotating sequences used as input for primer design.

**Conclusion:**

The accuracy of annotation by *Seq4SNPs *is significantly better than manual annotation (P < 1e-5).

Using *Seq4SNPs *to incorporate all annotation for additional SNPs and repetitive elements into sequences, for genotyping assay designer software, minimizes assay failure at the design stage, reducing the cost of genotyping. *Seq4SNPs *provides a rapid route for replacement of poor test SNP sequences. We routinely use this software for assay sequence preparation.

*Seq4SNPs *is available as a service at  and  , currently for human SNPs, but easily extended to include any species in dbSNP.

## Background

A survey of single nucleotide polymorphism (SNP) and primer design software reveals several packages that align EST or genome sequences to discover SNPs [1-6]. SNP-VISTA visualizes SNPs from aligned genome sequences [7]. Other packages take a chromosome region then use recorded SNP genotypes, and additional information, to reduce the set of SNPs that need genotyping [[8,9] and references therein]. SNP information packages collect data on a SNP, given a Reference SNP ID (rs number) or chromosome position, and may predict protein disruption, and are presented as searchable database resources [10]. These include LS-SNP, an annotated database of human SNPs within genes, and CASCAD, a rat/zebrafish database of candidate developmental SNPs [11,12]. The Functional Element SNPs Database (FESD) lists human SNPs in promoters and UTRs, CpG islands, transcription start sites, polyAdenylation signals elements of genes, and provides fasta output of SNPs with flanking sequences [13].

Several web tools output fasta sequences for SNPs. The dbSNP website outputs multiple fasta sequences flanking SNPs, given the rs numbers, after a time [10]. SNPper outputs flanking sequences annotated for adjacent SNPs [14]. SNP-Flankplus takes one or more rs numbers as input, with sequence length (like Seq4SNPs) [15]. SNP500 Cancer is a resequencing project focusing on identified SNPs associated with cancer, so displays potentially novel SNPs in flanking sequences [16]. Repeat Masker takes a fasta DNA sequence and masks it for repetitive elements, while SNPmasker takes as input a chromosome position (region) or sequence and outputs a fasta sequence masked for adjacent dbSNP SNPs and repeat sequences [17,18].

Primer design programs, like Primer3, take standard fasta DNA sequences and output PCR primer pairs with pertinent information like melting temperatures [19]. BatchPrimer3 will design both PCR and genotyping allele detection primers [20]. SNPbox is a primer designer for uniform PCR amplification across multiple regions, utilizing Primer 3 and incorporating sequence masking: input is fasta sequence and annotation with SNPs in flanking sequences is not included [21]. Genotyping assay design software outputs PCR as well as genotyping primers. These include Taqman^®^'s Assay-By-Design FileBuilder ; SNP-IT, used in SNPstream^®^'s Autoprimer, and iPLEX (Sequenom) also optimize PCR primers for multiplexing [22-24]. The standard fasta format is not a suitable input for these proprietary genotyping assay design software packages, so reformatting is required (Table 1).

**Table 1:** Formatting comparison for three medium-throughput genotyping platforms

**Genotyping method**	**Taqman **[22]	**SNPstream **[23,26]	**Sequenom **[24]
**Assay design software**	File Builder Assay-by-Design	Autoprimer	iPLEX

**URL**			

**Assay name**	Letters, numbers, – (hyphen) only	Letters, numbers, – only	Letters, number, – only

**Space**	Tab	Space	Space

**No. bases each side**	200–600	200	200

**Allele [A/G]**	Common first	-	-

**Additional information <other>**	1 = 201 (assay position)	Masked sequence	-

**Adjacent SNPs**	N	IUPAC	Lower case

**Masking**	N	N	Lower case

**Masked sequence**	First sequence	Second sequence, not 25 bases either side of SNP	First sequence

The low-medium genotyping platforms Taqman^®^, Beckman SNPstream^® ^and Sequenom iPlex™ come with assay design software packages that have different input requirements: each takes a file containing multiple SNP sequences, one per line; each line begins with a name and is followed by a sequence, separated by a space or tab, succeeded by either the position of the assay SNP (Taqman^®^) or any masked sequence (SNPstream^®^) or nothing (iPLEX™). Additional SNPs flanking the SNP to be assayed can also cause suboptimal assay performance if designed into an oligonucleotide binding site. Proprietary assay design software avoids additional variations, if the input sequences are suitably annotated by changing the variant nucleotide in the sequence to N, to the IUPAC code or to lower case. Basic sequence formats are summarised in the table, and follow the convention:

**Name <space> sequence [SNP]sequence <space> <other>**.

Examples for annotation of a synthetic SNP where the flanking sequence contains a masked sequence (repetitive element shown as NNNNNN/catgga distal to allele) and one adjacent SNP (N/Y/c before allele):

e.g. for Taqman:

SNP-01_rs14352008 ATGCANCAGG [A/G]ATGNNNNNNCAGG 1=11

e.g. for SNPstream:

SNP-01_rs14352008 ATGCAYCAGG [A/G]ATGCATGGACAGG ATGCAYCAGG [A/G]ATGNNNNNNCAGG

e.g. for Sequenom:

SNP-01_rs14352008 ATGCAcCAGG [A/G]ATGcatggaCAGG

**Figure 1:**
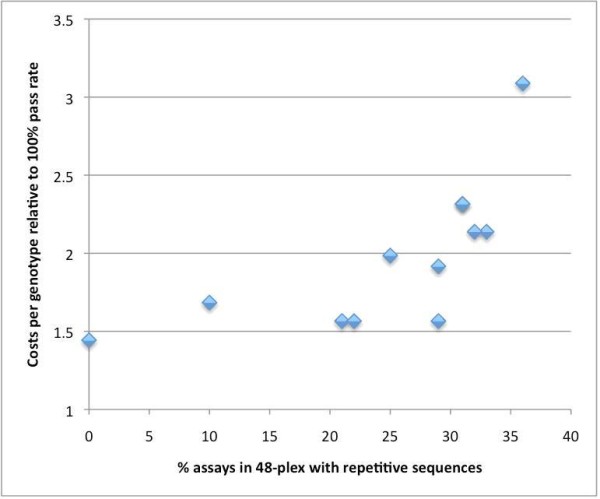
**Reduction of the cost of genotyping by reducing the number of sequences in a multiplex having repetitive elements**. The cost of genotyping using SNPstream 48-plex assays was calculated for a 100% pass rate (1 on y-axis): this cost increases non-linearly, as the pass rate decreases. Here we show how the cost increases with the number of assays containing repetitive sequences, per multiplex. Some repetitive sequences clearly affect the pass rate in the whole multiplex. We now use *Seq4SNPs *with Repeat Masker output, put masked sequences output from Seq4SNPs into SNP-IT primer design software for SNPstream, and replace SNPs rejected by SNP-IT. This improves assay success and reduces the cost of genotyping significantly.

Genotyping assay design software packages require as input a file containing specifically formatted DNA sequences, one line per assay (Table 1 and legend). To our knowledge, the only software (other than *Seq4SNPs*) that delivers sequences in genotyping assay designer format is Multiple SNP Query Tool (MSQT), used for *Arabidopsis *– but MSQT does not annotate the sequences for variations and repeats [4]. Seq4SNPs is unique in providing annotation of flanking sequences with adjacent SNPs from dbSNP, in assay designer formats. Successful genotyping assays avoid placing PCR or allele detection primers across a variation [25]. The performance of multiplexed genotyping assays decreases as the proportion of SNP sequences having repetitive elements increases (Fig. 1) [26]. Assay design software packages all utilize the information on variations and repetitive elements, if provided as annotation of the input sequences (Table 1). We identified the reformatting of the text and annotation of the flanking sequences for repetitive elements and variations as a rate-limiting step.

We required a sequence collection and formatting software which could take multiple inputs of one to fifty or more SNPs as rs numbers. When we began, ~300 SNP sequences were the required input for multiplex assay design software, which output optimally multiplexed groups of 48 SNPs, partly based on allele [23,26]. More flexible genotyping assay protocols now require fewer sequences. We also needed alternative inputs for newly identified SNPs without an rs number, i.e. chromosome position and allele.

**Figure 2:**
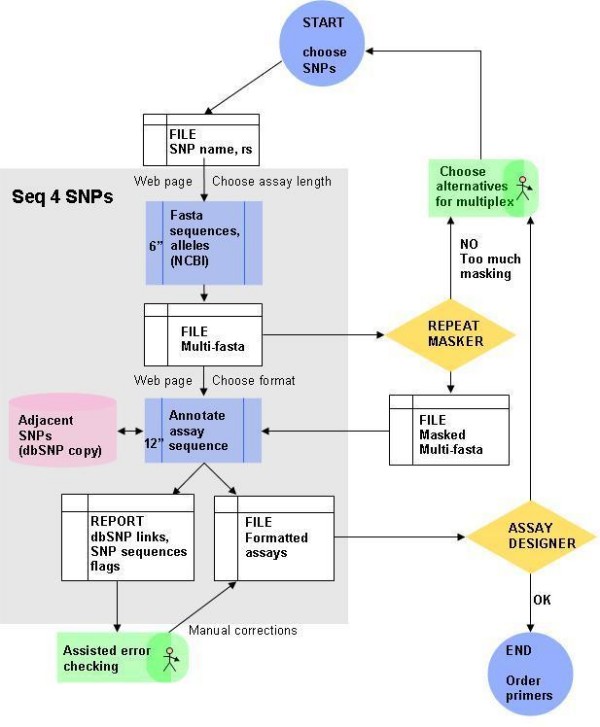
**Using Seq4SNPs software as part of the Workflow in SNPstream genotyping sequence preparation**. After choosing 48 or more SNPs to assay (START), input to *Seq4SNPs *software (grey box) is a text file (white, top) containing a list of rs numbers and assay names and the desired assay size (e.g. 200 nucleotides each side of the assay SNP). The first process (blue box, top) outputs a file of sequences in fasta format (white, centre). The user submits this to Repeat Masker which quickly produces a masked fasta file (white, right); at this point the user might reject some assays and start again if insufficient assays remain. In the second process (blue box, bottom) the *Seq4SNPs *fasta file is automatically used to annotate and reformat. The user inputs the masked file for this step. Final outputs (white, bottom) are a formatted sequence file for assay design software, and a report for Excel, containing warnings, error flags, minisequences for adjacent SNPs and links to dbSNP and is used for assisted error checking. User time is mainly confined to the delay points (green). The formatted assays are submitted to the SNP-IT at Autoprimer (Assay Designer), which rejects sequences that are insufficiently repeat-free, when the user may choose alternatives again (upward arrows). Legend: Seq4SNPs (grey box): times (sec ") are per SNP and are improving. Start and end points (circles); processes (blue rectangles); file/stored output (white rectangles); additional software decision aids (yellow diamonds).

We created *Seq4SNPs *for human SNP assay sequence preparation, to improve our medium-throughput genotyping workflow on SNPstream^® ^(Fig. 2). In our current workflow, about 48 sequences are prepared at a time, submitting fasta output of unannotated sequences to *Repeat Masker *. *Repeat Masker *outputs fasta sequence that is masked for repetitive regions. *Seq4SNPs *combines the masking from that file into the final formatted output, and adds additional variations (SNPs). A file containing all the sequences is submitted to the multiplex assay designer, which rejects SNPs with too much repetitive sequence. The user replaces rejected SNPs with alternatives having less repetitive sequence. One to three rounds of assay design are required per 48-plex. Thus, problematic assays are dealt with bioinformatically, before primers are ordered.

Automation of the sequence collection/annotation/reformatting process facilitates multiplex design iteration and dramatically reduces the time/cost involved in assay design (~10 seconds compared to 5–30 minutes *per SNP*). We have increased the success of assays and reduced the genotyping cost (Fig. 1). This paper describes the *Seq4SNPs *software, and the algorithms which collect, annotate and format SNP sequences.

Uses of *Seq4SNPs *software may be extended to other bioinformatics, as, given a list of rs numbers the software will retrieve new rs numbers, create fasta sequences, find the most common European allele, and find the gene and heterozygosity information, writing the data to output files designed for use in Excel, or to simple fasta text files with multiple sequences.

## Implementation

Briefly, *Seq4SNPs *takes a file of rs numbers, retrieves DNA sequences around the SNPs, from dbSNP, and formats them as a single file of fasta sequences, each with the allele in the fasta header. This fasta file is optionally submitted to *Repeat Masker *which outputs a masked fasta file. The original fasta file is automatically used for the *Seq4SNPs *annotation step, with the masked file (if submitted to *Seq4SNPs*). In the annotation step, the sequence is annotated with any dbSNP SNP or indel falling within the flanking sequences, and with masked sequence regions (if supplied). The annotated sequence is then formatted for assay designers. Completed sequences are saved to a file of sequences, formatted for submission to the genotype assay design software via the appropriate webpage (Table 1) [22,24,26]. A single SNP can be processed, also a SNP without an rs number. Data sources are listed (Table 2). A detailed description follows.

**Table 2:** Data sources

**Datum**	**Source**	**Notes**	**Step**
SNP rs or ss number*	User input	File or text input	1
Trivial name	User input	Same file as above	1
Size of assay sequence	User input	e.g. 200 specifies 200 nucleotides each side of assay SNP (401 altogether)	1
New rs number	NCBI dbSNP cluster page*	New rs retrieved when rs no longer in use** or if ss number submitted***	2
Fasta sequence, allele,	ditto	Fasta output with allele in header (major allele first)	2
Major allele, validation of assay, heterozygosity	ditto	'Allele' report.	
Fasta sequence (second attempt)	NCBI contig fasta sequence****	If sequence in cluster page too short: contig reference from cluster page*	2
Gene, chromosome	NCBI cluster page*	'Gene' report	2
Masked sequences	RepeatMasker (see text)	Takes fasta output above and produces fasta for next step.	3
Platform	User input	Choose TaqMan, SNPstream or Sequenom	3
Chromosome position, adjacent SNP list, with 21 nucleotide sequence etc.	Mysql local database with dbSNP data	Annotation of assay sequence using Seq4SNP algorithm	4
Validation, heterozygosity	Ditto	Part of **Adjacent SNP Report **(Fig 3) detailing each SNP and flagging placement mismatches	4
SNP assay sequences		Final output compatible with assay designers	4

Data used by *Seq4SNPs *is drawn from various sources, listed here: Seq4SNPs *inputs (italics)*, **outputs (bold)**. Some items are taken from web pages accessed by the universal resource locator (URL), or FTP download sites, shown below.

Example URLs:

*dbSNP rs cluster page:

**New rs number:  (if cluster page not available)

***rs number for ss:

****NCBI contig download:

**dbSNP downloads (human)**: fasta sequences and chromosome positions respectively from

**Note that to extend *Seq4SNPs *adjacent SNP addition to other species, data from other species may be downloaded **from the organisms folder and put into the MySQL database with the human SNPs

To begin, *Seq4SNPs *takes each SNP input as an rs number with a laboratory name. For batch processing it takes a set of assay SNPs in a plain text file, containing one SNP per line (e.g. 'TOX3-A1, rs12443621'). An intuitive user interface is provided for *Seq4SNPs*: the information or input file is submitted via a web page, where the user chooses the size of the flanking sequence (e.g. 200 for a 401 nucleotide sequence, with a minimum of 20 and a maximum of 600 nucleotides either side). Alternative starting points are: submitted SNP assay IDs (dbSNP ss numbers) instead of rs numbers; for a single SNP without an rs number, a sequence may be submitted, with chromosome, position and alleles, so that any sequence may be annotated [9]. Two other options are available: selection of 'major allele first' (e.g. [G/A] where G is the common allele) asks *Seq4SNPs *to retrieve the major Caucasian human alleles and to compile a report containing the heterozygosity data from the cluster pages; 'gene report' will retrieve gene name and description from each dbSNP cluster page. European common allele is selected from the data given on dbSNP cluster pages for AFD_EUR, or, if unavailable, from HAPMAP CEU. (For non-human species the allele is reported as given by the cluster page).

For each rs, *Seq4SNPs *automatically retrieves the flanking sequences and alleles from the NCBI cluster pages and creates one output file containing multiple fasta sequences with specialized headers for the annotation step. The sequence is taken from the HTML source code of the web page retrieved with a GET query (perl CGI). If a sequence is too short, it is retrieved from the NCBI contig sequence referenced by the dbSNP rs cluster page – the contig used is referenced by the first link listed under 'GeneView' if present, or under 'Integrated Maps', and the contig position utilized to request a sequence of appropriate length (URLS are listed on the webpage report, see example in Table 2 legend). If an ss is given or the rs is out of date, the new rs is first retrieved (Table 2 legend). *Seq4SNPs *outputs a text file containing fasta sequences (with customised fasta headers), which is used for the next stage, and may be saved. For identifying repetitive sequences this file can be submitted to the Repeat Masker server  which produces another fasta file containing the same sequences masked for repetitive sequences, homologous regions and transposon elements.

**Figure 3:**
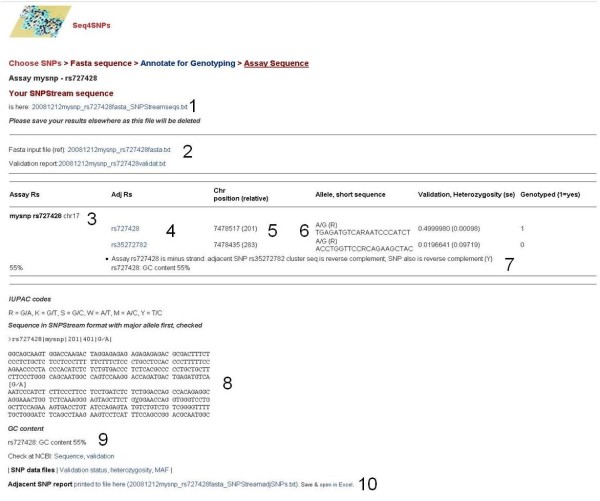
**Final output: web page from *Seq4SNPs *after processing one SNP, showing report**. (1) Link to file containing the formatted, linear assay sequence (not shown, as Table 1 legend). (2) Link to the fasta file (not shown, 50 nucleotides per line). (3) Report of SNPs for this assay, showing rs number, chromosome and name (if given): for each adjacent/assay SNP: (4) rs with link to dbSNP (NCBI); (5) chromosome position (and relative position in the assay sequence, numbered 1 – 401); (6) allele, IUPAC code for the allele and 21 nucleotide sequence; validation, heterozygosity and standard error. (7) Flag indicating how SNP sequences were matched, indicating that both adjacent and assay SNP sequences were reversed. Flags indicate complications in sequence matching, reporting both of the sequences which it attempted to match. (8) When a *single *SNP is requested, the assay sequence sequence is shown in a format useful for checking positions. (9) GC content is given.

The final stage of *Seq4SNPs *is sequence annotation and formatting for genotyping assay design software. Inputs are: the fasta file generated above (automatic) and, if repeat masking is required, the masked file from repeat masker is uploaded *via *the webpage. The assay format is selected as Taqman, SNPstream or Sequenom. A fasta file may be submitted as the primary input, here, provided that headers are compatible (see Fig. 3 for example). Seq4SNPs will generate an appropriate fasta header for any sequence input, here .

*Seq4SNPs *locates the chromosome position from the rs number, finds all SNPs within the assay flanking sequences, annotates the sequence text for these adjacent SNPs, adds the repeat/masked regions (optional), formats the sequence and creates an output text file, containing all annotated assay sequences, that is compatible with assay design software. Also, an 'adjacent SNPs' report file is supplied for Excel (and also shown as a web page), containing: links to dbSNP for all assay and adjacent SNPs; 21 bp sequence around every SNP or indel for checking; validation information; IUPAC code, chromosome position and position relative to the start of the assay sequence* [21,27]. GC content is calculated for SNPstream^® ^format. Warnings are given about SNPs (and repeats) very close to the assay position, or short sequences (Fig. 3).

Adjacent SNPs are most efficiently retrieved from a local MySQL database currently containing only human SNPs. (This dataset may easily be extended to other species by downloading the relevant data files: scripts and file locations are provided.) The database is updated periodically, providing the latest dbSNP output (currently 130). More frequent updates do not provide additional SNPs (Alternative sources of adjacent SNPs were unsatisfactory: querying the Ensembl MySQL database proved unreliable due to frequent down times; dbSNP cluster pages linked to adjacent SNPs only within genes.) Adjacent SNPs are retrieved by first obtaining the chromosome position for that rs number, then retrieving all SNPs within the flanking sequence by chromosome position plus and minus the assay 'size'. Orientation and exact position are checked by sequence matching across 21 bp (to ensure the correct IUPAC code is used for SNPstream^®^). Sequence matching tests for an exact match at two relative positions either side of the assay SNP, and in both orientations. If no match is found then the SNPs are displaced by one nucleotide either side of these positions, then by a second nucleotide, which resulted in correct placement for all SNPs we have tested. All unusual placements are flagged and the actual match sequences shown for manual checking. Flags indicate whether the SNP sequence (shown in the same sense as in dbSNP) is reverse complemented, or awkward placement due to: the adjacent SNP sequence being in opposite orientation to the assay sequence; chromosome positions apparently displaced by 1 or 2 nucleotides in repetitive regions; indels where one dbSNP sequence does not include the extra nucleotide(s), e.g. rs884013. Indels (e.g. [-/T] or [A/-]) are annotated by either replacing a nucleotide or added to the sequence (indicated by + in the report, at *). A flag for unsuccessful placement can be text-searched for (FUDGE or ERROR).

For multiple sequences, links to the output files are always given *before *processing, so that the user can watch progress, and recover the completed files. Every finished sequence is printed to the output file as soon as it is ready. Thus it is possible to process jobs containing more than 300 sequences: if a process does not complete then the input files can be truncated, and the remaining sequences completed. The 'adjacent SNPs' report can be recovered and opened in Excel, for assisted placement checking (21 mer sequences and links to dbSNP provided).

## Results

This software has been used in our Taqman and SNPstream workflow for four years, with incremental refinement. Approximate usage is currently about 40 SNPs per day. We routinely process more than 300 SNPs to fasta stage from a single input file. For a file of 327 fasta sequences, up to 227 were annotated in one go: the remainder were processed separately from a portion of the fasta file, for SNPstream Autoprimer. We have also generated large numbers of sequences for iPLEX. Single SNPs and multiples of less than 50 rs numbers are routinely processed for sequence preparation for Taqman and SNPstream. We also use *Seq4SNPs *for fasta retrieval for other bioinformatics tests and to determine the chromosome and position of a SNP given the rs number, or the latest rs for an old rs or ss number.

For 327 sequences submitted as a single batch, fasta retrieval took 4 seconds per SNP (6 minutes/100 rs numbers), with fast network and internet connections. Annotation with adjacent SNPs also took 4 seconds per SNP. Only a couple of seconds is hands-on time, which compares with manual sequence preparation at 5–20 minutes per sequence, excluding time taken to find adjacent SNPs outside of genes. User time was limited to seconds during processing, and afterwards a few minutes, checking the 'adjacent SNPs' report for the word 'ERROR' (in Excel or by HTML search) – which, if arising, might result in some manual edits; the 'adjacent SNPs' report also indicates any sequences that are 'TOO SHORT' and single-side matches (usually for indels) where the final sequence may be checked for correct placement against the 21 mer sequences given in the report.

To test the accuracy of annotation with adjacent SNPs, the assay designer sequence output from *Seq4SNPs *was compared, character by character, with a file containing correctly annotated and masked sequences for 327 assays. The test file of formatted sequences was generated for SNPstream by an earlier version of *Seq4SNPs*: the SNPstream format contains all the IUPAC codes for each SNP so that match orientation could be tested. We checked the sequences against dbSNP cluster pages and manually corrected for every adjacent SNP, verifying the correct IUPAC code and placement by sequence. (The original test file was submitted to Autoprimer and used in genotyping.)

We found that the automated placement gave a significant improvement over sequences corrected manually (P = 2e-6 by Fishers exact 2-sided test). This is based on a two by two table of correctly annotated vs. incorrectly annotated adjacent SNPs in 507 adjacent SNPs in 327 sequences prepared for the test batch: after manual correction, 20 adjacent SNPs were still misannotated, usually by use of the opposite IUPAC code and once by missing a SNP placed on the wrong side, while none were incorrect when done with *Seq4SNPs*. We also noted that since dbSNP had been updated from version 128 to 129, 73 additional SNPs, mostly indels, were now correctly placed into our sequences. Retrospective assessment of assay sequences delivering poor genotyping results with *Seq4SNPs *may rapidly reveal new variations not previously known to be in the flanking sequences.

The latest version of the software (September 2008) contained 0 error flags, indicating correct placement of 507 adjacent SNPs in 327 sequences. 380 sequences were correct for major European allele assignment. There were 177 flags for 327 sequences, of which 111 were reverse complement, 40 were single side matches and 12 were new placements 1 base from the given chromosome position (indels). The flags were confined to 96 SNPs, but none had placement *errors*.

## Conclusion

Thorough testing has shown that *Seq4SNPs *delivers multiple sequences suited to genotyping assay designers, that are accurately annotated with adjacent SNPs. Repeat masking annotation and Caucasian major allele recovery are also completed correctly. Automated annotation by Seq4SNPs is significantly more accurate even than manual verification over large numbers of sequences, and by using *Seq4SNPs *with Repeat Masker to eliminate poor test SNPs, we have improved our SNPstream genotyping efficiency from a 70% pass rate to greater than 80%.

## Availability and Requirements

The software is currently a web service available with adjacent SNP addition for human SNPs. Source code will be made available on request.

Software was written in Perl CGI and accessed on a server via internet web pages. It was deployed on a dual-processor server with Intel Pentium 4 chips (3.4 GHz each with 1024 Kb cache) and 1 GB RAM running Debian 4 OS4 linux  and Apache 2 , and is now on an HP Proliant ML115 server with dual core G5 Opteron 1214 2.2 GHz chips, 2 G RAM, running Ubuntu 5.0.51a-3ubuntu5.1 server  with Perl v5.8.8 and MySQL 5. Client-side (user) testing was done using the Firefox web browser  or Safari v. 3 or Explorer (version 6 but NOT version 7) on PCs running Mac OSX Jaguar and Windows XP/2000. To obtain data for all SNPs quickly, the dbSNP tables (Table 2 legend) detailing SNP chromosome positions, alleles and reference sequences were placed into two tables in a MySQL database  on the server, using Perl scripts.

## Abbreviations

SNP: Single Nucleotide Polymorphism; rs: Reference SNP ID (dbSNP); ss: NCBI Assay ID (dbSNP); CGI: Common Gateway Interface; URL: universal resource locator or web address; IUPAC: International Union of Pure and Applied Chemistry.

## Authors' contributions

HIF created and tested the software and wrote and revised the manuscript. PP provided substantial input into the format of the manuscript and figures. AD corrected the flow of the manuscript and provided information on SNPstream workflow. SS provided the data on improvement of genotyping and the file of manually checked test sequences, CL the data on cost reduction, while both provided critical input into the design and function of the software and testing its output. CB tested the software within the genotyping workflow and taught other testers to use it. JM provided perl scripts and populated the first MySQL database and is the manager of the external server. DFE is essential for continuing maintenance of the project. All authors read and approved the final manuscript.

## Authors' information

HIF is a DPhil (PhD) in biochemistry and an MSc in information technology and has held the post of Senior Research Associate within the genotyping group of the Department of Oncology, funded by Cancer Research UK, since 2005. DFE is a Principal Research Fellow and PDPP is Senior Clinical research Fellow of Cancer Research UK. AD holds the post of Senior Research Associate at the Department of Oncology, CL is a Chief Technical Officer.
